# Immunovirotherapy: The role of antibody based therapeutics combination with oncolytic viruses

**DOI:** 10.3389/fimmu.2022.1012806

**Published:** 2022-10-13

**Authors:** Mahdie Jafari, Maryam Kadkhodazadeh, Mina Bahrololoumi Shapourabadi, Nasser Hashemi Goradel, Mohammad Ali Shokrgozar, Arash Arashkia, Shahriyar Abdoli, Zahra Sharifzadeh

**Affiliations:** ^1^ Department of Immunology, Pasteur Institute of Iran, Tehran, Iran; ^2^ Department of Molecular Virology, Pasture Institute of Iran, Tehran, Iran; ^3^ HUM Immune Biotech Company, Tehran, Iran; ^4^ Department of Medical Biotechnology, School of Advanced Technologies in Medicine, Tehran University of Medical Sciences, Tehran, Iran; ^5^ National Cell Bank of Iran, Pasteur Institute of Iran, Tehran, Iran; ^6^ School of Advanced Medical Technologies, Golestan University of Medical Sciences, Gorgan, Iran

**Keywords:** oncolytic virotherapy, cancer immunotherapy, nanobody, antibody, combination therapy, immunovirotherapy, T cells, Nk cells

## Abstract

Despite the fact that the new drugs and targeted therapies have been approved for cancer therapy during the past 30 years, the majority of cancer types are still remain challenging to be treated. Due to the tumor heterogeneity, immune system evasion and the complex interaction between the tumor microenvironment and immune cells, the great majority of malignancies need multimodal therapy. Unfortunately, tumors frequently develop treatment resistance, so it is important to have a variety of therapeutic choices available for the treatment of neoplastic diseases. Immunotherapy has lately shown clinical responses in malignancies with unfavorable outcomes. Oncolytic virus (OV) immunotherapy is a cancer treatment strategy that employs naturally occurring or genetically-modified viruses that multiply preferentially within cancer cells. OVs have the ability to not only induce oncolysis but also activate cells of the immune system, which in turn activates innate and adaptive anticancer responses. Despite the fact that OVs were translated into clinical trials, with T-VECs receiving FDA approval for melanoma, their use in fighting cancer faced some challenges, including off-target side effects, immune system clearance, non-specific uptake, and intratumoral spread of OVs in solid tumors. Although various strategies have been used to overcome the challenges, these strategies have not provided promising outcomes in monotherapy with OVs. In this situation, it is increasingly common to use rational combinations of immunotherapies to improve patient benefit. With the development of other aspects of cancer immunotherapy strategies, combinational therapy has been proposed to improve the anti-tumor activities of OVs. In this regard, OVs were combined with other biotherapeutic platforms, including various forms of antibodies, nanobodies, chimeric antigen receptor (CAR) T cells, and dendritic cells, to reduce the side effects of OVs and enhance their efficacy. This article reviews the promising outcomes of OVs in cancer therapy, the challenges OVs face and solutions, and their combination with other biotherapeutic agents.

## 1 Introduction

Cancer is rapidly becoming the leading cause of mortality worldwide. Every year, nineteen million new malignancies are diagnosed, resulting in about ten million deaths (1, 2). Because cancer is a complicated and heterogeneous disease with numerous genetic mutations, current cancer therapies frequently do not achieve the desired outcomes for the majority of malignancies, despite many promises of progress in treatment. Consequently, cancer treatment has become a challenge, so more efficient treatment procedures are required (3, 4). Traditional treatments, such as surgery, chemotherapy, hormone therapy, and radiation therapy, have not only unfavorable adverse effects on individuals in the majority of patients but also yield minimal long-term benefits (5).

Immunotherapy has been a promising approach to cancer treatment over the last two decades. It is target-specific, can be adjusted to the needs of each patient, and has fewer side effects than earlier cancer therapies. Immunotherapy drugs can be more effective against cancer when combined with other therapies, such as radiation therapy, chemotherapy and targeted drugs. As an example, several studies have shown promising results of using a mix of chemotherapy and immunotherapy as a first strike against non-small cell lung cancer (2, 5, 6). To date, various immunotherapeutic approaches have been introduced in cancer treatment, such as pro-inflammatory cytokines, cancer vaccines, adoptive T-cell therapy, antibody-based immunotherapies, and oncolytic viruses (OVs) (7, 8).

Currently, oncolytic virotherapy (OVT) is one of the most popular cancer immunotherapy approaches owing to the flexibility of viral production platforms and providing a multimodal strategy to selectively and efficiently target and destroy tumor cells (9, 10). Furthermore, OV platforms could be applied without depth knowledge of tumor antigens in various malignancies (11). OVs provide multi-mechanistic therapeutic effects against the majority of cancer types, but like with many other current cancer therapies, oncolytic virotherapy still faces challenges and hurdles before becoming an effective anticancer therapy (3). Despite some encouraging outcomes, OVT still is not completely effective in most cases because of some issues such as tumor bulk penetration, anti-viral immune responses, and unfavorable tumor microenvironment (TME) (12, 13). On the other hand, due to off-target infection and sequestrations by non-specific tissues, especially in systemic administration, there are some safety concerns about using OVs as therapeutic agents (14).

Despite this, in clinical trials of monotherapy, OVs with older generations of armings (such as GM-CSF) have elicited a potent and robust response. Newer methods, like combining OVs with immunotherapies to turn “immune-cold” tumors into “immune-hot” ones, can almost certainly make OVs more effective (3, 15, 16). The use of rational combination therapies and targeting have been raised to improve the efficacy of OVs, and these combinations may integrate multiple methodologies and technologies that can increase patient benefit from the treatment (9, 17).

Oncolytic viruses have been used in combination with other cancer treatment modalities of immunotherapy or cell therapy, such as antibodies, nanobodies, bispecific (antibody-based immunotherapies), checkpoint inhibitors, adoptive T-cell therapy, natural killer (NK) cells, and T-cell engagers (BiTE), to improve cancer treatment (10). In this review, the challenges of OVT are discussed in detail, including its low efficiency, safety issues, and delivery methods, and finally, we focus on combining OVs with other biotherapeutic strategies to overcome the challenges.

## 2 Intro to virotherapy: From concept to bedside

### 2.1 An overview of virotherapy

The concept of employing viruses to treat cancer cells has existed almost as long as viruses have been discovered (18, 19). For more than a century, viruses have been considered potential cancer-fighting agents (20–22). Since the middle of the 1800s, case reports indicated that spontaneous microbial infections in cancer patients might sometimes temporarily reduce tumor burden and, thus, many therapeutic trials have been conducted employing wild-type non-attenuated viruses in cancer therapy (22–25). In the late 1890s, a finding that a “flu-like” condition accompanied by generalized inflammation corresponded with a reduction in tumor cells in a leukemic patient further confirmed the potential therapeutic significance of viruses, in particular (22, 26). In another case, the measles virus has been shown to be an effective natural anticancer agent in the treatment of Burkitt’s lymphoblastic lymphoma (27).

For a long time, the development of selective and harmless viruses was impeded by a lack of tools for viral genome modification (16, 28, 29). It took a few decades for OVT to reach its full potential when recombinant DNA technology became widely used to increase safety (6, 22, 29). The use of a thymidine kinase (TK)-negative mutant of Herpes Simplex Virus (HSV-1) as a possible treatment for gliomas was the first report of a virus modification to reproduce only in dividing cells. TK-mutated HSV-1 has been demonstrated to reproduce preferentially in cancer cells (30–32). A mutated adenovirus (Ad), dl1520 (also known as ONYX-015), was discovered in 1996 that had the E1B55K gene deleted (33, 34). Since the E1B-55kD gene product can bind to and inactivate p53, it was assumed that the deletion of E1B-55kDa renders the mutant adenovirus unable to inactivate p53 in normal cells and, therefore, the viral replication cycle would not be completed. Moreover, the replication of ONYX-015 might be related to the indirect inactivation of the p53 pathway in tumor cells due to the loss of upstream regulators such as p14ARF (35). Nevertheless, it was shown that the p53 status can not impose a restriction on ONYX-015 replication. Actually, the loss of E1B-55K-mediated late viral RNA export results in inability of ONYX-015 to replicate in normal cells. Since, the tumor cells have a special capacity to efficiently export late viral RNA in the absence of E1B-55K, ONYX-015 would selectively replicate in cancer cells (36). As a result, clinical uses for OVs are increasingly prominent due to technological advances (37, 38).

OVs can selectively reproduce in cancer cells and propagate throughout a tumor without affecting the healthy tissues (39, 40). Despite some viruses’ natural tropism for tumors, the wide range of tumor forms and histologic origins makes it challenging to link OVs to a specific malignancy (41, 42). Additionally, it is crucial to consider the tumor-specificity, possible pathogenicity, immunogenicity, druggability, and the viral stability while choosing a virus.

The administration of OVs, either systemically or locally, in cancer-bearing hosts successfully induces antiviral immunity. As a result, OV treatments activate two separate immune responses: antiviral and anticancer. While antitumor immunity is advantageous, antiviral immune responses, including innate and adaptive, are thought to be harmful to the success of OV-based therapy. Indeed, it is conceivable that antiviral immune responses might impede strong viral replication and spread, reducing direct oncolysis of cancer cells,and therefore the efficiency of OV therapy (43). As a result, the most effective “time window” for most OVs to activate anti-tumoral immunity is within the first 1-2 weeks of administration, before the virus is eliminated. One of the major challenges of OV immunotherapy is to strike a balance between the desirable induction of new anti-tumoral immunity and the competing anti-viral immunity while preventing undesired antiviral effector processes from becoming the dominant response pathway, thereby obstructing the acquisition of acquired anti-tumoral immunity. Because of this, researchers are now looking into a number of ways to treat anti-OV immune responses (44). Many studies are developing strategies to enhance OVs construction, reduce clinical toxicity, design efficient OV delivery systems, and increase efficacy by utilizing contemporary genetic engineering approaches (45). A large number of OVs are being investigated in clinical studies, and an even greater number are being evaluated in preclinical studies. The safety of virotherapy has been shown by clinical researches utilizing various OVs to treat different cancers (42, 46, 47).

### 2.2 Viruses that have already received regulatory approval for the treatment of cancer

Following 30 years of research and encouraging findings from several clinical trials, the OV has attracted a lot of interest, leading to an OV approved by FDA for cancer treatment (25, 38). Four OVs have been approved for use in the treatment of various malignancies. Despite being licensed in Latvia, the first OV, a picornavirus named Rigvir, was never widely used worldwide (48, 49). In 2005, the Chinese SFDA approved the use of a modified Ad, known as Oncorine (H101), in combination with chemotherapy for the treatment of head and neck cancer (50–52). Talimogene laherparepvec (T-VEC, Imlygic), an attenuated HSV containing granulocyte-macrophage colony-stimulating factor (GM-CSF), was approved by the FDA in October 2015 for the treatment of melanoma in the US (53–56). In Japan, a modified version of the HSV, called Delytact, received time-limited and conditional marketing approval for the treatment of malignant gliomas in 2021 (21, 26, 57–59). A summary of the aforementioned OVs has been presented in **Table 1**.

**Table 1:** Global-approved oncolytic viruses (OVs).

Product	Country approved	Approval year			Virus type	Modification	Dosage
**DELYTACT** **(teserpaturev/G47D)**	Japan		2021		HSV Type I	G207’s 47 gene and US11 promoter deletion	1×10^9^ PFU
**Imlygic^®^** **(talimogenelaherparepvec)**	United States and Europe		2015		HSV Type I	HSV1 gamma 34.5 and ICP 47 deletion and expressing GM-CSF	1×10^6^- 1×10^8^ PFU
**Oncorine (H101)**	China		2005		Adenovirus serotype 5	E1B-55k and E3 deletion	5×10^11^-1.5×10^12^ VP
**Rigvir (ECHO-7)**	Latvia		2004		Picornavirus	_	TCID50 10^6^/mL

VP, Virus Particle; TCID50, Median Tissue Culture Infectious Dose; PFU, Plaque Forming Unit.

### 2.3 Action mechanisms of oncolytic viruses: From cytolysis to microenvironment modulation and antitumor immunostimulation

**Figure 1:**
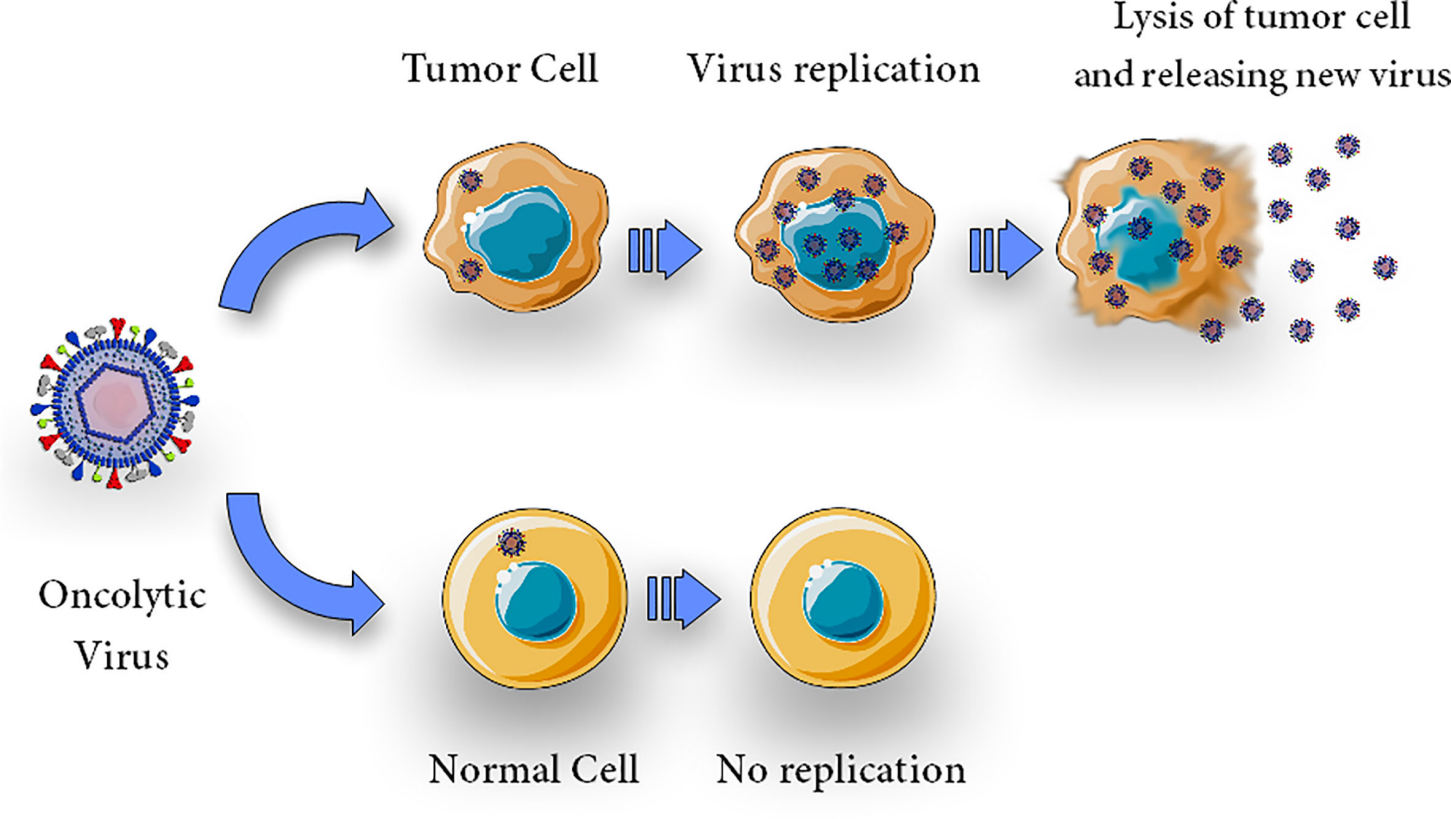
Direct cytolytic activity. Oncolytic virus can selectively infect, replicate, lyse and kill cells. Upon infection with an oncolytic virus, the oncolytic virus replicates in tumor cells and causes oncolysis but does not harm normal cells.

Tumor cells, due to their resistance against apoptosis, appear to be a preferred breeding ground for a wide variety of viruses (22, 60). Viral infection kills tumor cells by several mechanisms including direct cytolytic activity which is thought to be its main oncolytic mechanism; this activity is due to the OVs’ capacity to selectively infect, replicate, and kill cancer cells (**Figure 1**). It is now generally accepted that virotherapy’s efficacy can be attributed to a number of different processes, including alterations in the tumor’s micro-and macroenvironment and intricate immune control (40, 61, 62). OVs are cytolytic due to viral propagation and host cell bursting (63), and the viral infection may trigger apoptosis in the host cell (20)

Lysed tumor cells produce endogenous danger-associated molecular patterns (DAMPs), tumor-associated antigens (TAAs), virus-derived PAMPs, and immune-stimulatory cytokines, triggering the anti-tumor immune responses (64, 65).

The key and distinguishing characteristic of OVs is their selective amplification and replication in cancer cells, leading to the death of tumor cells without affecting the normal ones. The intensity of their anti-tumor action depends on the modalities of OV-induced cancer cell death (66, 67). Immunovirotherapy, also known as OV immunotherapy or viroimmunotherapy implies an OV infection that causes an inflammatory TME by eliciting anti-tumor immune responses. Furthermore, there is evidence that OVs have the capacity to transform an immunologically “cold” TME into a “hot” one *via* the production of chemokines and cytokines. It is worth noting that a balance between helpful anti-tumor immunity and harmful anti-virus immune responses is necessary for optimizing immunovirotherapy (68, 69). In order to modify the TME, OVs may also target tumor-associated stroma cells, such as endothelial cells. Immunogenic cell death (ICD) can be caused by OVs, which promote endoplasmic stress, resulting in the release of DAMPs, such as ATP, HMGB1, ectocalreticulin, and pro-inflammatory cytokines (64). STING, TLR1, and TLR3 on immune cells sense PAMPs and DAMPs, establishing a pro-inflammatory microenvironment that stimulates the production of pro-inflammatory cytokines such as type I IFNs, interleukin (IL)-1, IL-6; TNF-a, GM-CSF, and chemokines such as CCL2, CCL3, CCL5, and CXCL10 (70, 71), leading to transformation of immunologically “cold” T-cell into “hot” T-cells (72, 73). Neutrophils and macrophages are attracted to the site of infection by CCL3 and CXCL10 chemokines, which are involved in anti-cancer responses. Aggregation of PAMPs with NK cell virus-recognition receptors causes early NK cell influx (74). Activated cytotoxic NK cells may produce cytolytic components and activate FAS-FASL, killing virus-infected cells. NK cells emit IFNs and TNF-a to excite macrophages, DCs, and T-cells. This activation of NK cells and DCs induces them to produce IFNs, TNF-a, IL-12, IL-6, and chemokines, which work both autocrinely and paracrinely to increase the initial innate response (21, 75, 76).

The tumor-specific T-cell response is the foundation of adaptive immunity against tumor cells during OV infection. Antigens presented in the context of an MHC molecule, co-stimulatory molecules, and cytokines are required for antigen presenting cells (APCs) to activate antigen-specific T-cell responses successfully (77). The released TAAs and neoantigens following tumor cell lysis by OVs are processed by APCs and are presented on their surface with MHC molecules to CD4^+^ and CD8^+^ T-cells (78). Also, OV-infected cells or mature APCs release various cytokines and chemokines, which aid in the recruitment and reactivation of T-cells. Both stimulated T-cells and B-cells could promote tumor regression and are capable of eradicating distant or freshly transplanted tumors without relying on an OV (79, 80).

In addition to tumor cells, the tumor’s extracellular matrix (ECM) and vasculature are also affected by OVs. More than 60% of a solid tumor’s mass comes from the ECM, which is a non-cellular compartment made by activated cancer-associated fibroblasts (CAFs). Collagenous matrix, proteoglycans, and hyaluronan build up in the ECM, providing an impenetrable and stiff barrier around cancerous cells. Because of these physical impediments, OVs have a tough time reaching the entire tumor mass (21). Ilkow et al. showed that interaction between CAFs and cancer cells improves vesicular stomatitis virus (VSV)-based therapies (61).

**Figure 2:**
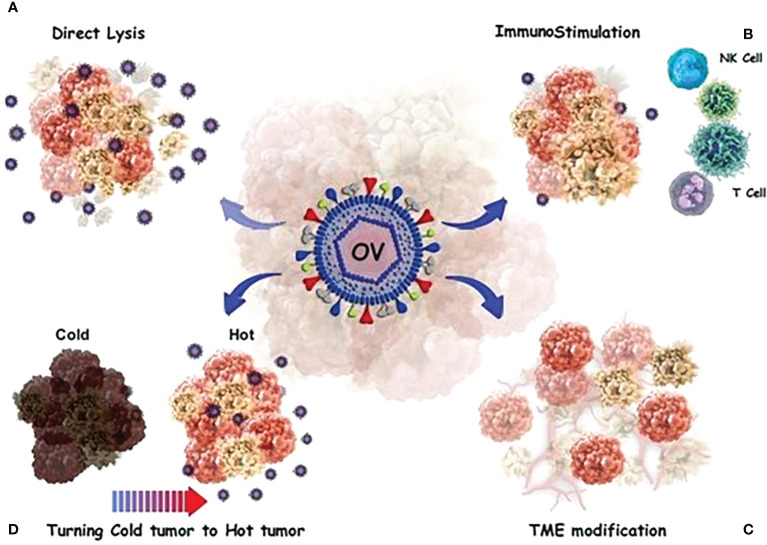
OVs have a direct or indirect toxic effect on tumor cells. **(A)** Direct oncolysis; The viruses can infect cancer cells and then replicate until the cancer cells rupture. The newborn viruses are then released to infect more cancer cells. **(B)** Neoantigens and debries from lysed cancer cells activate and recruit dendritic cells (DCs) into the tumor microenvironment and T cells migrate to the site of infection. **(C)** a number of processes occur, including the alteration of the tumor’s micro-and macro-environment and the control of the immune response in a complex modulation. **(D)** OVs stimulate innate immunity and turn “cold” tumors into “hot” tumors by stimulating immune cell recruitment and activating systemic anticancer adaptive immunity to reduce tumor growth.

In contrast, tumor cells release transforming growth factor-beta 1 (TGF-1), which promote OV infection in CAFs. Tumor cells produce large quantities of fibroblast growth factor 2, making them vulnerable to viral infection. It has been reported that OAd not only could lyse glioblastoma cells, but also kills glioblastoma-associated stromal cells (81). **Figure 2** provides a detailed anti-cacner mechanism of action of OVs.

## 3 Various types of OVs

There are two broad classes of OVs: 1) viruses that have a natural tropism for cancer cells; the naturally cancer-selective OVs utilize the abnormal signaling pathways that support their growth in cancer cells; and 2) those engineered specifically to replicate only in cancer cells (16). The activity of OVs reflects their underlying biology and the host-virus interactions that have evolved in the struggle between pathogenesis and immunity (29). The lack of an anti-viral response in cancer cells is an important mechanism of tumor selectivity for both categories. Interferons (IFNs) are secreted by normal cells in response to viral infection after intracellular pathogen recognition receptors identify viral RNA, DNA, or proteins (PRRs). Hundreds of effector genes, called IFN-stimulated genes (ISGs), are expressed as a result of this signaling cascade and aid in the elimination of the viral infection (16). Myxoma virus (MYXV; poxvirus), Newcastle disease virus (NDV; paramyxovirus), reovirus, Seneca Valley virus (SVV; picornavirus), measles virus (MV; paramyxovirus), poliovirus (PV; picornavirus), vaccinia virus (VV), Ad, HSV, and VSV are some examples of oncolytic viruses (**Table 2**) (63, 66, 82). **Table 2** lists the different types of viruses that have been used for oncolytic purposes.

**Table 2:** Characteristics of major oncolytic viruses.

Virus	Poxvirus 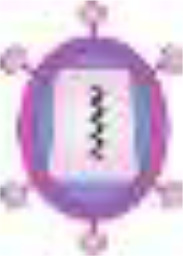	NewcastlediseaseVirus(NDV) 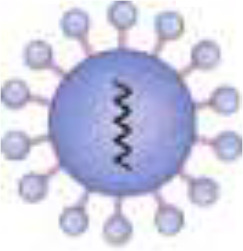	Reovirus 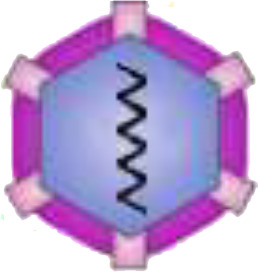	Measles virus 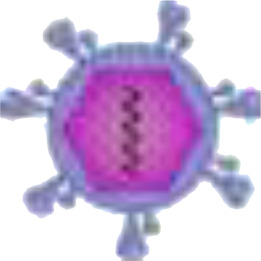	Herpes simplex virus 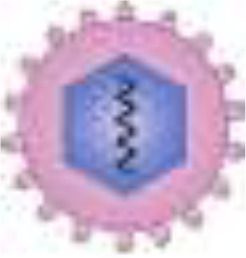	AdenoViruses 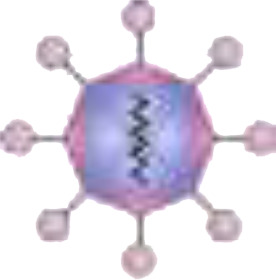	Parvovirus 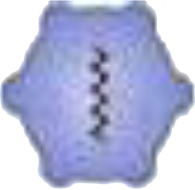	Poliovirus 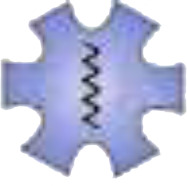	Vesicular Stomatitis Virus 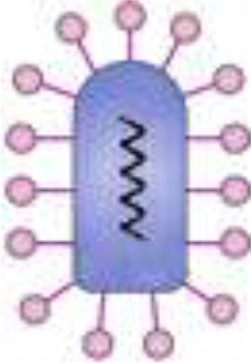	Seneca Valley Virus 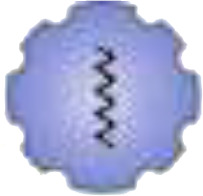
**Genome** **(Size)**	dsDNA (160–190 kb)	SS (–) RNA (15 kb)	dsRNA (23 kb)	SS (–) RNA (16 kb)	dsDNA (154 kb)	dsDNA (35 kb)	ssDNA (5 kb)	SS(+) RNA (7.5 kb)	SS (–) RNA (11 kb)	SS (+) RNA (7 kb)
**Virion**	Complex	Enveloped	Naked	Enveloped	Enveloped	Naked	Naked	Naked	Enveloped	Naked
**Replication** **Site**	Cytoplasm	Nucleus	Cytoplasm	Nucleus	Nucleus	Nucleus	Nucleus	Cytoplasm	Cytoplasm	Cytoplasm
**Receptor**	Heparan, laminin, Chondroitin,integrin-1, CD98	Sialic acid	Sialic acid, JAM1	SLAMF1 (CD150), CD46, Nectin 4	HVEM, Nectin 1, Nectin 2	CAR	Sialic acid residues, P antigens	CD155	LDLR	Anthrax toxin receptor 1

### 3.1 Natural tumor-replicating viruses

#### 3.1.1 Poxviruses

One of the most important OV platforms now showing promising outcomes in clinical studies is the vaccinia virus (VV), a member of the Poxviridae family that naturally attacks malignancies (21). Tumors are a recognized target of VV strains owing to the activation of the epidermal growth factor receptor (EGFR) pathway in malignant cells (83). Researchers showed that vascular endothelial growth factor A (VEGF-A) enhances oncolytic VV cytotoxicity by studying the effect of hypoxia on VV infection (84). Tumor-derived VEGF increases VV internalization, leading to enhanced replication and cytotoxicity in both tumor cells and normal respiratory epithelial cells in an AKT-dependent manner (85). Moreover, tumor cells lack the anti-viral cytokines that protect normal cells from viral infection because of their poor interferon (IFN) response. Multiple attenuated VV mutants have been developed to improve tumor-specific targeting and safety in normal tissues (86, 87). The Pexa-Vec (JX-594), an oncolytic VV armed with GM-CSF and disruption of the TK gene, is under investigation in phase I and II clinical trials for treating renal cell carcinoma, advanced breast cancer, and advanced soft-tissue sarcoma (NCT03294083 and NCT02630368). Furthermore, an engineered vaccinia OV, RGV004, encoding a bispecific CD19/CD3 antibody, is in phase I clinical trials for the treatment of refractory/relapsed B-cell lymphoma (NCT04887025). A phase I/II clinical trial showed that Pexa-vec intratumoral injection was safe and effective in treating surgically incurable metastatic melanoma (NCT00429312).

#### 3.1.2 Newcastle disease virus

The Newcastle disease virus (NDV), belonging to the family Paramyxoviridae, is an enclosed virus and contains negative-sense single-stranded RNA (88, 89). The HN protein interacts with sialic acid receptors on the surface of host cells to bind tumor cells, and when the activated F protein joins the viral and host cell membrane, the HN protein fuses with the virus (90). As a result, the virus’s genome penetrates the cytoplasm of the host. NDV can also enter cells by endocytosis and clathrin-mediated endocytosis (91). There is evidence that gene-editing technologies make it simple to introduce foreign genes with anti-tumor activities into the extensive genome of NDV (92). Numerous clinical investigations have shown that NDV has a very excellent safety profile for patients and has considerable anti-cancer activity (93). For instance, because NDV only affects the type I IFN-deficient glioblastoma cells, an inhibitor of IFN signaling eliminates the NDV resistance in type I IFN-positive cells (8, 94). In two clinical studies (NCT03889275 and NCT04613492), the drugs Durvalumab (anti-PD-L1) and attenuated NDV with the GM-CSF and IL-12 genes (MEDI5395 and MEDI9253, respectively) are being used. NDV with durvalumab, is in phase I clinical trial for treating advanced solid tumors (95).

#### 3.1.3 Reovirus

Reovirus (RV), an unenveloped virus containing a double-stranded RNA belongs to the Reoviridae family (96). The oncolytic properties of wild-type reovirus are due to the virus’s preference for replicating in cancer cells (97). Reovirus has the ability to kill cancer cells because of its preferential ability to multiply in cancer cells. Ras overexpression impairs the PKR (protein kinase RNA-activated) pathway, allowing reovirus to infect tumor cells preferentially (98–100). Reolysin (also known as Pelareorep) (101), serotype 3 RV, is the well-known oncolytic RV that as a single agent or in combination with other therapeutic strategies (29), is under investigation in clinical trials (NCT04102618, NCT04445844, and NCT04215146).

#### 3.1.4 Measles virus

The measles virus (MV), which belongs to the genus Morbillivirus in the Paramyxoviridae family, is an enveloped virus containing negative-sense single-stranded RNA (102). Three receptors, CD46, SLAM/CD150, and poliovirus-receptor-like-4, are used by MV to infect host cells (103, 104). However, CD46 is not a tumor-selective receptor because of its expression on normal cells. MV is a hopeful OV candidate due to its good safety profile, which includes the absence of dose-limiting toxicities and spontaneous oncotropism (21, 104, 105).

Heinzerling et al. performed the first Phase I dose-escalation test with a live MV, Edmonston-Zagreb vaccine strain against cutaneous T-cell lymphoma (CTCL) (106). Clinical trials using a measles virus that expresses the human sodium/iodide symporter SLC5A5 are currently being conducted (107). The Mayo Clinic (USA) has launched a number of Phase I/II clinical studies (NCT00390299, NCT02364713, NCT02068794, NCT02700230, NCT01503177, NCT01846091) to examine the clinical safety and usefulness of MV-CEA and MV-NIS (95).

#### 3.1.5 Picornaviruses

Picornaviruses have promising anti-cancer effects in patients (108). Picornaviruses are tumor-specific due to the overexpression of their entry receptors on cancerous cells, including CD155, integrin a1b2, intercellular adhesion molecule-1 and/or decay-accelerating factor (CVA21), anthrax toxin receptor 1 and sialic acids and anthrax toxin receptor 1 (109). Clinical studies using oncolytic picornaviruses typically go smoothly, and no off-target infections have yet been reported. Intratumoral administration of the oncolytic poliovirus PVSRIPO, the live attenuated, type I poliovirus (Sabin) vaccine harboring an internal ribosome entry site (IRES) of human rhinovirus type 2, has demonstrated initial promise in patients with recurrent glioblastoma multiforme (NCT03712358) (110). In contrast, PVSRIPO infects macrophages and DCs in culture, causing the expression of major histocompatibility complex class II (MHC II) and the generation of IFN-b and IL-12 (9, 87).

### 3.2 Genetically engineered (modified) oncolytic viruses

#### 3.2.1 Herpes simplex virus

Talimogene laherparepvec (T-VEC; Imlygic), the first OV presently licensed by the FDA, is a member of the Herpesviridae family (56). In addition to T-VEC, other HSV-based OVs have been developed, such as G47δ, oHSV-IL12, G207, and rRp450 (111, 112). The majority of HSV-based vectors carry deletions in ICP34.5, a neurovirulence gene that restricts virus replication to tumor cells overexpressing the Ras gene (113). The inactivation of the ICP6 gene, which encodes a viral homolog of the cellular ribonucleotide reductase (RR), is another mechanism of HSV specificity (114, 115). The mutant virus replication is limited to actively proliferating cancer cells with high levels of RR because this enzyme is necessary for creating deoxyribonucleotides (29). The results of a Phase Ib study using T-VEC in combination with the CTLA4 checkpoint inhibitor, Ipilimumab, in people with advanced melanoma were published by Puzanov et al. (116). Additionally, the combination of OV with nivolumab has showed very promising results (12, 116–118).

#### 3.2.2 Adenoviruses

Adenoviruses (Ads), non-enveloped viruses with icosahedral capsid and double-stranded linear DNA genomes, are members of the Adenoviridae family, specifically the genus Mastadenovirus. The capsid of Ads contains three major proteins, including Hexon, penton-base, and fiber proteins, which give them specific tropism characteristics (119). In clinical research, adenovirus serotype 5 (Ad5) is the most frequently utilized viral vector (120). Ad5 penetrates the targeted cells *via* interacting its fiber knob protein with coxsackievirus and adenovirus receptors (CARs) (118, 121).

DNX-2401 (Delta-24-RGD; tasadenoturev) is an oncolytic adenovirus, replication -competent adenovirus. A 24-base pair deletion in the E1A gene promotes tumor selectivity by preventing viral replication in normal cells with a functioning Rb pathway. An RGD-motif was added to the fiber H-loop to boost potency, allowing the virus to enter cells through v3 or v5 integrin. On tumor cells, especially glioma stem cells, these integrins are abundant (122). In preclinical models, DNX-2401 kills glioma cells through direct oncolysis and by inducing immunological responses against tumor antigens, resulting in long-term antitumor immunity and tumor regression. DNX-2401 is now being tested in clinical studies for the treatment of recurrent glioblastoma (NCT03896568) (123). Also, additional studies are being conducted to examine its effectiveness against recurrent gliomas when used in conjunction with other treatments, such as checkpoint inhibitors. In one active phase II study, DNX-2401 was injected directly into a recurrent glioblastoma or gliosarcoma followed by pembrolizumab every 3 weeks for up to 2 years or until disease progression (NCT02798406) (38).

H101 is an adenovirus with an E1B deletion that has been approved in China for the treatment of nasopharyngeal carcinoma. H101 was tested in a randomized Phase III clinical study with 160 people who had advanced squamous cell carcinomas of the head and neck or esophagus (124). The patients were randomly assigned to chemotherapy (cisplatin and 5-FU for chemotherapy-naive patients, or adriamycin and 5-FU for patients who had previously received platinum chemotherapy) with or without H101 (5× 10^11^ to 1.5 ×10^12^ viral particles per day by intra-tumoral injection) for five consecutive days every three weeks. A total of 123 patients completed treatment, and were able to be evaluated for response. Patients who received cisplatin/5-FU + H101 exhibited a response rate of 78.8%, compared to 39.6% in the cisplatin/5-FU-only cohort. Patients who got the adriamycin/5-FU and H101 virus, as well as the adriamycin/5-FU -only group, both achieved a 50% response rate; however, these groups had a limited number of participants (n = 18). There was a substantial difference in response rate between all patients who got H101 and individuals who only received chemotherapy. The most common adverse events were fever, injection site reactions, and flu-like symptoms. Based on these findings, the Chinese regulatory agencies approved H101 in combination with chemotherapy for the treatment of nasopharyngeal cancer (10). In addition to the aformentioned viruses, several additional viruses, including parvovirus, poliovirus, vesicular stomatitis virus, Seneca valley virus, have been engineered to be used as OVs in combating cancer (21). **Table 3** summarizes the types of viruses that are in different phases of clinical trials.

**Table 3:** Summary of clinical trials of monotherapy and combination therapy of oncolytic viruses.

	Oncolytic virus	Combination therapy	Cancer	Dosage	Clinical phase	Clinical trial No
**Adenovirus**	Ad-p53	Nivolumab or Pembrolizumab	Head and Neck Squamous Cell Carcinomas; Colorectal Cancer; Hepatocellular Carcinoma	5 x 10^11^ VP single dose	I/II	NCT02842125 NCT03004183
	Ad-CEA	Avelumab	Colorectal Cancer	1 x10^11^ VP 6 doses	II	NCT03050814
	Ad-MAGEA3	Pembrolizumab	Non-Small Cell Lung	2 x10^11^ VP single dose	II	NCT02879760
	ONCOS-102	Pembrolizumab	Melanoma	3 x10^11^ VP 3 doses	I	NCT03003676
	LOAd703	Atezolizumab	Malignant Melanoma	1 x10^9^ VP 12 doses	I/II	NCT04123470
	Ad-TK	Pembrolizumab/ valacyclovir33/SBRT	Non-small Cell Llung Cancer; Triple-negative Breast Cancer	5 x 10^11^ VP single dose	II	NCT03004183
	H101	Camrelizumab	Recurrent Cervical Cancer	1.5 x10^12^ VP 2-6 doses	II	NCT05234905
	CG0070		Bladder Cancer	1 x10^12^ VP 3-9 doses	II	NCT02365818
**Herpes simplex virus**	T-VEC	Pembrolizumab	Melanoma	1×10^8^ PFU	III	NCT02263508
	OH2		Pancreatic Cancer	1×10^7^ CCID50 6 dose	I/II	NCT04637698
	T-VEC	Nivolumab/Trabectedin	Sarcoma	1×10^7^ PFU	II	NCT03886311
	HF10	Ipilimumab	Metastatic Melanoma	1×10^7^ TCID50 6 doses	II	NCT03153085
	OrienX010	Pembrolizumab	Melanoma	3 × 10^11^ VP 3 doses	I	NCT03003676
	OH2		Advanced Bladder Cancer	1×10^7^ CCID50 single dose	II	NCT05248789
**Newcastle disease virus**	MEDI5395	Durvaluma	Advanced solid tumor	dose-expansion study to assess the safety	I	NCT03889275
**Vaccinia virus**	MVA-p53	Pembrolizumab	Solid tumor	5.6 × 10^8^ PFU 3 doses	I	NCT02432963
	JX-594		Metastatic Hepatic Carcinoma	1×10^8^-3×10^8^ PFU single dose	I	NCT00629759
	TG4010	Nivolumab	Non-small cell lung cancer	1×10^8^ PFU single dose	II	NCT02823990
	Pexa-Vec (JX-594)	Tremelimumab/ durvalumab	Refractory Colorectal cancer	3 × 10^8^ PFU 4 doses	I/II	NCT03206073
		Ipilimumab	Advanced solid tumor	1×10^9^ PFU 5 doses	I	NCT02977156
		Sorafenib	Hepatocellular Carcinoma	1 × 10^9^ PFU 3 doses	III	NCT02562755
	Olvi-Vec	Bevacizumab/ cisplatin	Ovarian Cancer	1×10^9^ PFU single dose	III	NCT05281471
	TBio-6517 (Rival-01)	Pembrolizumab	Solid tumor, Colorectal cancer	multiple doses	I/II	NCT04301011
	OVV-01		Advanced Solid Tumors	1 × 10^12^ VP single dose	I	NCT04787003
**Vesicular stomatitis virus**	VSV-IFNβ-NIS	Pembrolizumab	Non Small Cell Lung Cancer Neuroendocrine Carcinoma	5×10^10^ TCID50 single dose	II	NCT03647163
**Reovirus**	Reolysin	Pembrolizumab	Advanced pancreatic	4.5 × 10^10^ TCID50	II	NCT03723915
		Atezolizumab	Breast	4.5 × 10^10^ TCID50 4 doses	I	NCT04102618
			Sarcomas Metastatic to the Lung	3×10^10^ TCID50 5 doses	II	NCT00503295

VP, Virus Particle; TCID50, Median Tissue Culture Infectious Dose; PFU, Plaque Forming Unit; CCID50: Cell Culture Infectious Dose 50%.

As a result of genetic engineering, a wide variety of potentially pathogenic viruses have been manipulated for safety and tumor-targeting applications in the past two decades. Genetic modifications including the deletion of viral genes, the use of transcription regulatory elements such as promoters and enhancers, and the alterations of viral surface proteins have been widely used to increase the effectiveness of targeted OVT (82, 125).

## 4 Tumor targeting strategies of oncolytic viruses

Despite the remarkable preclinical success of OVT, clinical applications remain limited. One of the most significant challenges that must be overcome is viral targeting. Various strategies have been established to achieve targeting OVs toward tumor cells. As previously described, some viruses, such as reovirus and NDV have an intrinsic tropism for tumor cells, whereas the other ones, such as Ad and HSV, should be adapted or engineered to be cancer-specific (126). Virus adaptation to cancer cells is frequently accomplished based on cancer cell modifications, including self-sufficiency in growth signals, resistance to apoptosis, neoantigen expression, and an unlimited replication potential that can be used for OVs selective infection and killing of cancer cells (127). In this regard, different approaches have been used to direct OVs into cancer cells, including modifying the virus’s surface (transductional targeting), introducing specific genes downstream of specific tumor promoters or inserting genetic elements into virus genomes such as miRNA and siRNA to boost OV specificity (transcriptional targeting), and deleting virus genes that are required for replication in normal cells but have little effect on reproduction in cancer cells (33, 128, 129).

### 4.1 Transductional targeting

Detargeting viruses from their normal cells and retargeting them to a specific cell is a critical step in designing OVs, especially for adenovirus-based oncolytic viruses. As previously stated, Ads as one of the most utilized viruses in cancer treatment, have no innate tropism for cancer cells, whereas they exhibited a broad range of tropism for normal cells due to CAR expression in the majority of normal cells. As a result, an unaltered virus can infiltrate and harm normal cells by systemic injection. Hence, the vector’s inherent tropism should be eliminated to reduce possibly detrimental side effects. Scientists usually use two methods to solve the problem: adding ligands like peptides, antibody fragments, and nanobodies to the structure of the virus, and using bispecific adaptors. Van Erp et al. coupled transcriptional targeting by utilizing a tumor-specific promoter with transductional targeting by using an anti-CEA nanobody incorporated into Ad. CXCR4E1.B2 virus capsids. They showed that using a single specific domain for CEA which was inserted genetically into the Ad fiber could improve the specificity of infection and the ability of Ads to reproduce in cancer cells (130) Another method for modifying the surfaces and tropism of OVs is pseudotyping. This strategy is often done by replacing coat proteins with similar proteins from related serotypes, leading to a new tropism without changing the balance of the genome. In this regard, adenoviral fiber protein pseudotype switching is a reasonable strategy for transductional retargeting. Owing to the upregulation of CD46 on many malignant tumors, researchers replaced Ad5 fiber with the fiber of serotypes 11/35 to target tumor cells (131, 132). Another strategy that has been developed to target viruses toward tumor cells, as previously mentioned, is the use of bispecific adapters. Adaptors are molecules with two ends that bind to the viral proteins and the receptors on the cancer cells. This strategy’s main advantage is the ability to use multiple adaptors to attach to the same vector without affecting the vector`s structure. Due to the overexpression of the high molecular weight melanoma-associated antigen (HMWMAA) on melanoma cells, Curiel et al. designed a bispecific adaptor, scDb MelAd, to target Ad to melanoma cells selectively. They demonstrated significantly reduced infectivity (> 50-fold) of capsid mutant Ads, restored (up to 367-fold increase), CAR-independent and HMWMAA-mediated infectivity of these mutant viruses by scDb MelAd specifically in melanoma cells, compared to a vector with wild-type fibers (133). Additionally, a universal platform for Ad5 detargeting and retargeting using the SpyTag and SpyCatcher system was developed and demonstrated that Ad5 efficiently wasredirected into VEGFR2-expressing cells using an adoptor incorporating SpyCatcher and an anti-VEGFR2 nanobody (under publication data).

### 4.2 Transcriptional targeting

The effectiveness of transductional techniques has been inadequate for realizing the full promise of virotherapy in the clinic. For instance, early gene therapy experiments used therapeutic genes driven by viral promoters, such as the CMV promoter, which caused non-specific damage in normal cells and tissues as well as cancer cells. However, the use of tumor-specific promoters, which are overexpressed in tumors, stimulates the particular expression of therapeutic genes in a certain tumor, boosting their localized action and reducing mislocalization side effects (134). TTF-1 promoter, glypican-3 protein (GPC3), human secretory leukocyte protease inhibitor (hSLPI), Mucin 1 (MUC1), cyclooxygenase 2 (COX2), epithelial glycoprotein (EPG2), and human telomerase reverse transcriptase (hTERT) are the most common tumor-specific promoters used in transcriptional targeting (7). For instance, combining transcriptional targeting using the tissue-specific SLPI promoter and transductional targeting with the ovarian cancer specific adaptor protein, sCARfC6.5, which contains the coxsackie-adenovirus receptor ectodomain and a single-chain antibody specific for c-erbB-2, increased transgene expression in ovarian tumors while decreasing expression in normal tissues, including the liver, in comparison to single-approach targeting (135). As abovementioned, inserting micro-RNAs (miRNAs) into the virus genome is a new way to improve their specificity and reduce off-target effects. Many teams have used the differential landscape of miRNA expression between normal and malignant cells to hinder OVs proliferation in healthy cells. In one study, it has been demonstrated that adding several copies of a miR-124 recognition sequence into the 3′UTR of the oncolytic HSV-1’s crucial ICP4 gene prevents the virus from infecting normal cells. This phenomenon occurs because of the high expression of miR-124 in healthy neurons but not at all in glioblastoma cells (136, 137). In another study, Luo et al . employed a triple-regulated OAd containing miR143, survivin, and RGD to improve the effects of OAds. They showed that when Ad-RGD-Survivin-ZD55-miR-143 was introduced into cells, it could inhibit cell growth, migration, and invasion, as well as halt cells in the G1 phase and induce cell death (138).

Despite the use of many techniques for targeting viral vectors in cancer virotherapy and boosting the virus’s efficacy in cancer treatment, when the virus must be injected systemically for the treatment of metastatic malignancies, this treatment strategy encounters a number of challenges (125) that must be overcome before the systematic administration of OVs to improve their anti-tumor activities (126). The most common challenges of delivering the virus through the bloodstream are viruses’ identification as foreign agents and elimination from the body before they can reach the tumor site, known as immune-mediated clearance (127), and virus sequesteration by non-specific tissues such as the liver, lungs, and spleen (128). On the other hand, tumors are high-pressure settings with a dense and disorderly collection of cells due to thick stromal tissue and limited lymphatic drainage (42). To address these issues, scientists have designed a variety of approaches, which are described in more detail below.

### 4.3 Solutions to the challenges of OVs’ systemic delivery

Complement activation, pre-existing immunity, or the release of inflammatory cytokines (IL-6, IL-12, and TNF) in response to vectors all contribute to OV clearance by the immune system. Some OVs, including vaccinia and HSV-1, produce anti-complement components to evade the immune system (139). HSV-1 secretes glycoprotein E, which functions as an IgG Fc receptor and efficiently inhibits IgG Fc-mediated complement activation as well as antibody-dependent cellular cytotoxicity (ADCC) (140). Pre-existing immunity canoccur due to the virus’s ubiquitous nature (Ad and Reovirus), previous vaccination (vaccinia and MV), or earlier oncolytic viral treatment (141). There are currently various solutions being tested for these issues. Changing the surfaces of viral vectors by shielding with polymers such as (poly ethylene glycol (PEG), poly L-lysine, and *N*-[2-hydroxypropyl] methacrylamide (HPMA)) and lipidic vesicles often reduces their immunogenicity and increases vector persistence in the bloodstream (142). Cellular carriers, in which cells are taken from a model organism that has been infected and put back in, are another way to deliver OVs. Immune cells, stem cells, and tumor cells have been used to generate experimental OV cell carriers. Among cell carries, stem cells, according to *in vitro* and *in vivo* studies, are the most outstanding candidates for systemic delivery of OVs since they allow viruses to infect the target cells and replicate in, conceal them from the immune system, and target tumors (143, 144). In this regard, Mader et al. used mesenchymal stem cells (MSCs) to efficiently deliver oncolytic MV to ovarian cancer and protect the virus from neutralizing anti-viral antibodies. They found that using MSCs as carriers increased their localization and infiltration into tumors and transferred oncolytic MV infection to tumors, leading to enhancing mice survival (143). Immune cells, especially DCs and T-cells, have been used successfully in pre-clinical research to transfer several OVs to tumors. For instance, DCs infected with reovirus have been shown to efficiently transport and deliver their oncolytic payload into melanoma cells, even in the presence of neutralizing antibodies (145). On the other hand, OVs are removed from the bloodstream by mononuclear phagocytic cells, splenic macrophages, and hepatic Kupffer cells in the spleen and liver following systematic administration. This clearance frequently occurs following the decorating of viral particles with antibodies and complement proteins or their interaction with coagulation factors (126). However, some viruses, such as Ads, may bind directly to scavenger receptors on Kupffer cells, resulting in the release of pro-inflammatory cytokines, which may cause severe toxicities (146). The answers to these challenges are fairly similar to the methods outlined for immune response escape. In case of Ads, the hexon protein, as the most frequently structural protein, has a critical role in liver sequestration through interaction with coagulation factor IX and scavenger receptors (147, 148). Different strategies have been developed to avoid this sequestration, such as genetic alteration in the hypervariable region (HVR) of hexon (149), pseudotyping (complete change of HVR) (150), and pharmacological agents (such as warfarin and protein obtained from snake toxin and factor X-binding protein) (151, 152). Surface PEGylation is a popular strategy for reducing non-specific tissue absorption. Kwon et al. detected a substantial 10^5^ increase in tumor to liver ratio when Ad was treated with a PEGylated chitosan specific to the folate receptor compared to naked Ad (153).

### 4.4 Intratumoral spread of OVs in solid tumors

Tumor physiology is a major issue in cancer treatment since tumors come in a variety of forms and sizes, making it difficult to predict how and where medications, such as OVs, will be absorbed (154). Therefore, viruses transferred within the tumor can only infect and spread cells near blood vessels, leaving the rest of the tumor untreated. Therefore, researchers have focused on establishing mechanically activated transport mechanisms to promote OVs penetration and increase virus anti-cancer activity (155). Most solutions for this barrier rely on virus-encoded matrix-degrading enzymes and anti-fibrotic agents. Diop-Frimpong et al. showed that the penetration and effectiveness of intratumorally injected oncolytic HSV were improved by using Losartan, which is a clinically approved angiotensin II receptor antagonist with anti-fibrotic effects (156). In another experiment, Guedan et al. created a replicating Ad capable of producing soluble sperm hyaluronidase (PH20) (AdwtRGD-PH20). Intratumoral AdwtRGD-PH20 treatment caused hyaluronan (HA) degradation, enhanced viral dispersion, and tumor regression occurred in all of the treated tumors (157). In addition, expressing matrix metalloproteinases-1 and -8 in oncolytic HSV increased viral dispersion and treatment efficiency by breaking down tumor-associated sulfated glycosaminoglycans (158).

## 5 Oncolytic virotherapy in combination with cancer immunotherapeutics

Although various studies demonstrated viruses potential in eliminating tumor cells, there is currently no report that virotherapy can lead to a complete cure of cancer alone due to the previously mentioned challenges. However, there is ample evidence that OVs can be considered as the basis of combination therapy in different cancers due to their multiple mechanisms of action and their simultaneous effects on tumor cells, immune cells, and the TME (45, 159). Here, we have discussed possible combinations of OVs with different biological products that can overcome monotherapy challenges and limitations in cancer treatment.

These combinations can be classified as 1) Armed recombinant oncolytic viruses that carry the coding sequence of other therapeutic agents which are excellently discussed elsewhere by Kontermann (69), and 2) Combining OVs with other biologic therapeutics separately.

**Figure 3:**
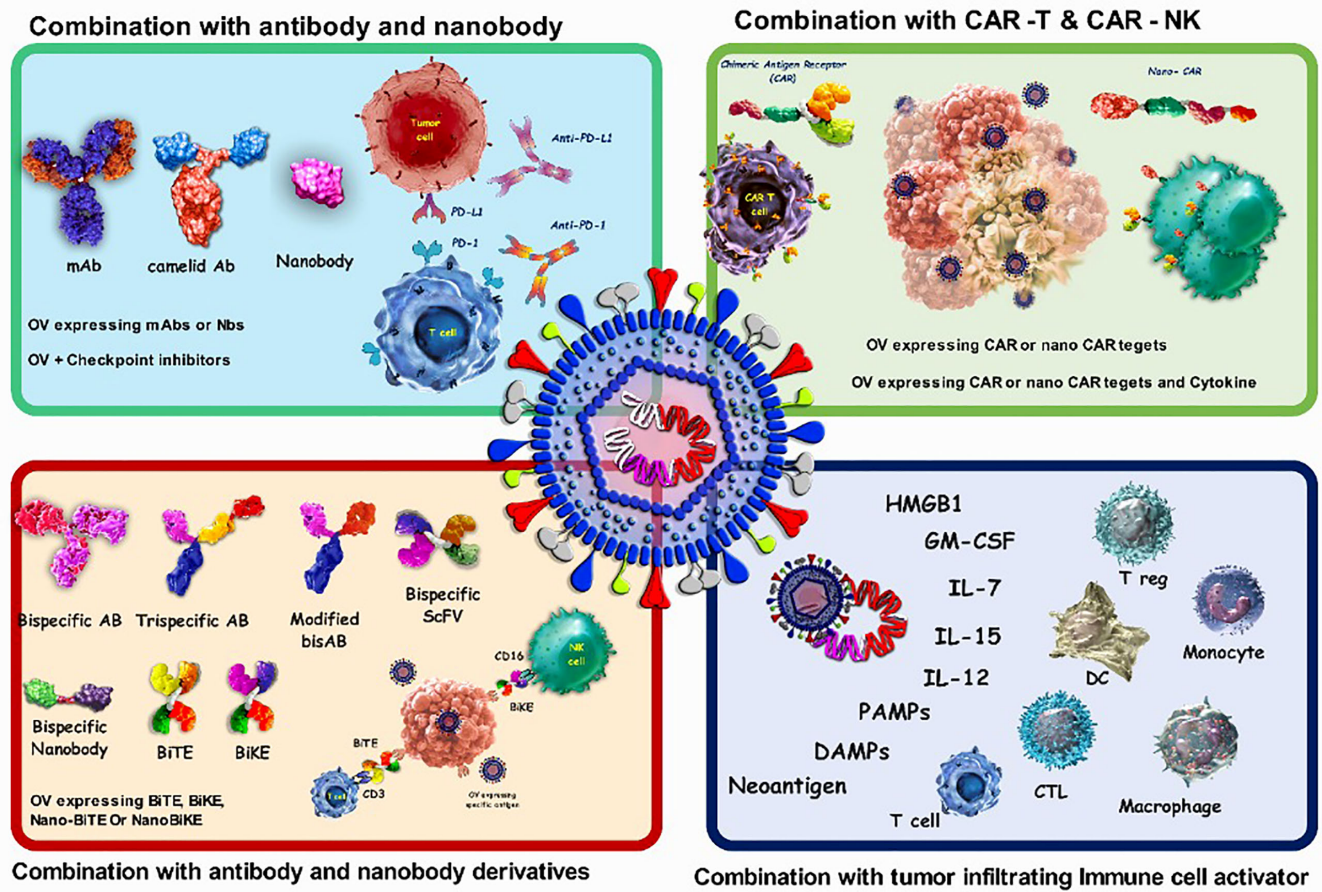
Characteristics of oncolytic virus combination therapy. OVs attack and destroy tumor cells preferentially. Lysis of tumor cells releases neoantigen, PAMPs which trigger PRRs, which then produce inflammatory cytokines and antiviral type I IFNs. Viruses can activate cell death pathways, resulting in immunogenic cell death phenotypes such as necroptosis, pyroptosis, immunogenic apoptosis, and autophagic cell death. Antibodies that target cell surface indicators of immune cells (checkpoint inhibition), cancer cells (targeted therapy), or both (bispecific antibodies) are wellestablished in cancer therapy. combination of oncolytic viruses with antibody and CAR-T cells; CAR-T cells bind to the antigen on the surface of tumor cells and kill them, but they cannot migrate deeper into the dense tumor mass to remove antigennegative tumor cells. also, CAR-NK cells show more anticancer activity than CAR-T cells because they attach to stress ligands on the surface of tumor cells. The oncolytic virus attacks and destroys tumor cells, eliminating the tumor's dense structure.

Combining viruses with either the coding sequence or the final protein form of antigen binding biologics)such as antibodies, nanobodies and CAR-T cells(can help viruses overcome some limitations, such as possible off-target side effects and non-specific uptake (**Figure 3**). Antibodies (Abs) are widely used in targeted therapies and mostly recognize TAAs on tumor cells. Abs are also combined with OVs to improve possibility of attachment of the virus to its target cells. As a proof to this claim, combining OVs with immune checkpoint inhibitor (ICI) antibodies is leading to promising anti-cancer results. Despite this issue, one of the main limitations of antibodies, especially for the treatment of solid tumors, is their poor tumor penetration due to physical barriers. This can be improved by using smaller antibody fragments, such as single-domain antibodies, scFv, and Fab. There are also other functional antigen-binding therapeutic formats with some notable advantages that are used in some other studies in the viro-antibody therapy field that can be translated into the clinical trial. These promising structures can be combined effectively with OVs for better therapeutic efficacy. For improving the combinational therapy outcome, nanobodies can also be used to not only assist OVs in specific targeting, but also helping viruses for more efficient penetration into solid tumors. **Table 4** summarizes the various strategies of combination therapy with oncolytic viruses.

**Table 4:** Summary of combination therapies with oncolytic viruses and other immunotherapeutic agents.

	Virus	Strategies for Antibody Gene Expression	Format	Target	Result
Combination oncolytic virus with antibody (ICI, mAb,nanobody)	Ad (Ad5/3-E1AΔ24)	Replacement of early genes (E3) with Ig chains	IgG2	Human CTLA-4	Subcutaneous xenograft mouse tumor model/intratumoral virus injection: OV-encoded antibody was detected in xenografts; 43-fold higher antibody concentration in tumor versus plasma; 81-fold higher antibody concentration detected in tumors after injection of antibody-encoding OV compared with antibody-encoding replication-deficient control virus (160).
	Influenza A virus (IAV)	Heavy chain in PB1 segment downstream of PB1 gene *via* 2A; light chain in PA segment downstream of PA gene *via* 2A	IgG and scFv	IgG and murine CTLA-4	Antibody insertion reduced titer, replication and *in vivo* morbidity and mortality of IAV Functions of OV-produced IgG was similar to hybridoma-produced Ab. Subcutaneous syngeneic bilateral mouse tumor model/intratumoral OV application: scFv-encoding OV showed superior tumor growth inhibition (both flanks) and prolonged survival compared with parental virus (161).
	HSV-1	Separate transcription unit, MMLV LTR promoter	scFv fused to mouse IgG1	Murine CTLA-4	Bilateral subcutaneous syngeneic mouse tumor model/low dose intratumoral OV injection of right flank tumor: antibody-encoding OV increasesd tumor growth inhibition of injected (117).
	NDV	Additional transcription unit downstream of P gene	scFv	Murine CTLA-4	Intradermal syngeneic mouse tumormodel/irradiation/intratumoral OV injection: antibody-encoding OV + X-ray showed similarly increased survival and tumor growth inhibition than parental virus + X-ray + systemic α-CTLA-4 when compared with α-CTLA-4 alone (162, 163).
	MV	Separate transcription unit downstream of H gene	scFv-IgG1 Fc fusion	Murine CTLA-4, murine PD- L1	Subcutaneous syngeneic mouse tumor model/intratumoral OV injection: α-CTLA-4-encoding OV reduced tumor progression, whereas α-PD-L1- encoding OV prolonged survival both compared with control virus. Both antibody-encoding OVs increased T cell infiltration, decreased Treg infiltration and resulted in splenocyte activation (164).
	VSV	Additional transcription unit between G and L genes	scFv	Human PD-L1	Subcutaneous syngeneic mouse tumor model with hPD-L1-expressing mouse tumor cells/intratumoral OV injection: Antibody-encoding-OV or combination of parental OV + intraperitoneal scFv reduced tumor growth and improved survival in comparison to monotherapies, Increase of activated CD8+ T cells in spleen of mice cured after treatment with antibody-encoding-OV compared with normal mice (165).
	Vaccinia virus	Additional transcription unit with viral H5 promoter	scFv	Human PD-L1	cell lines and activated T cells were infected: parental OV resulted in translocation of PD-L1 to cell surface in cancer cells; antibody-encoding OV delivered sufficient α-PD-L1 scFv to block cell surface detection of PD-L1 on cancer cells; OV-encoded scFv increaseed granzyme B production and prevented OV-induced decrease in perforin release by T cells (166).
	Ad (EnAd, chimeric type B Ad)	Replacement of early genes (E3) by Ig chains linked *via* IRES	IgG1	Human HER2 (Trastuzu- mab)	OV-encoded antibody showed direct antitumor activity and triggers ADCC *in vitro.* Subcutaneous xenograft mouse tumor model/intratumoral virus injection enhanced antitumor efficacy of antibody-encoding OV compared with parental virus or trastuzumab for Her2-positive xenografts. Higher tumor-to-blood antibody concentrations by antibody-encoding OV compared with conventional antibody application (167).
	NDV (wt velogenic Italien strain)	IgG heavy and light chains as separate, adjacent additional transcription cassettes with gene stop and gene start signal for viral transcription	IgG	CD147 (metuximab)	Orthotopic xenograft mouse tumor model/intravenous OV application: antibody- encoding OV resulted in antibody expression in tumors and tumor necrosis. Reduced intrahepatic metastasis and prolonged survival compared with parental OV (168).
	Vaccinia virus (GLV- 1h68: Lister vaccine strain, triple mutant)	Separate transcription unit, viral promoters (SEL, SL [VEGF] or SEL+SL)	nanobody	VEGF (scFv) + EGFR (nanobody); VEGF (scFv) + cross-species FAP (scFv)	Subcutaneous xenograft mouse tumor model/intravenous OV injection:OVs encoding single antibodies (targeting EGFR, VEGF, or FAP) inhibited tumor growth more rapidly (one xenograft model) or stronger (other xenograft model) than control virus. OVs encoding two antibodies resulted in strongest tumor growth inhibition, significantly superior to control virus; significance not reached in comparison to single antibody-encoding OVs (169).
OVs plus bispecific antibodies	HSV-1 (G207)	Inserted as separate transcription unit with CMV promoter	BiTE or nanobody- scFv fusion	Human PD-L1 (scFv or nanobody) × human CD3 (scFv)	PD-L1-positivity of T cells did not prevent expansion or effector functions after activation by purified BiTE: Co-cultures of infected tumor cell line, PBMC-derived T cells and immunosuppressive ascites fluid: BiTE-encoding OVs, not control virus, induced depletion of tumor cells (170).
	Ad (EnAd, chimeric type B Ad)	Inserted as separate transcription unit with CMV promoter	BiTE	Human folate receptor-β × human CD3	Ex vivo ascites model with total ascites cells: BiTE-encoding OV induced T cell activation and expansion, depletion of macrophages, and increase of M1 markers on remaining macrophages (repolarization) superior to parental and control viruses (171)
	MV	Additional transcription unit downstream of H gene	BiTe	Human CEA × murine or human CD3, human CD20 × murine CD3	Subcutaneous syngeneic mouse tumor models/intratumoral OV injection: BiTE-encoding OV resulted in (i) prolonged survival, in one of two models superior to control virus or direct BiTE injection; (ii) increased T cell infiltration and activation; and (iii) protective immunity (to ental tumor cells not expressing the BiTE-target. Thus indicative of antigen spread, i.e., activation of endogenous T cells specific for tumor antigens) (172).
	Vaccinia virus	Separate transcription unit, late viral promoter	BiTe	Human EphA2 × human CD3	Co-cultures of infected tumor and unstimulated T cells or PBMCs: BiTE- encoding OV, not control virus, induced T cell activation, which depended on presence of EphA2-positive cells, and T cell-dependent bystander tumor cell killing. Lung metastasis xenograft mouse tumor model/intravenous OV and/or PBMC injection: BiTE-encoding OV showed significantly delayed tumor growth compared with controls (173).
OVs plus CAR-T Cell and CAR-NK Cells	Ad	HER2 chimeric antigen receptor specific cytotoxic T lymphocytes	scFv	Metastatic HER2 Positive Solid Tumors	HER2 chimeric antigen receptor specific cytotoxic T lymphocytes (HER2 specific CAR-T cells), in combination with intra-tumor injection of CAdVEC, an oncolytic adenovirus that was designed to help the immune system including HER2 specific CAR-T cell reacted to the tumor. https://clinicaltrials.gov/
	Vaccinia virus	CD19-expressing oncolytic virus CF33-CD19	scFv	eradicate solid tumors	The combination of CD-19-directed CAR-T with CD19-encoding OV resulted in greatly improved survival of mice compared to antigen-mismatched combinations. https://www.imugene.com/
	HSV-1	IL15/IL15Rα sushi domain fusion protein	scFv	Glioblastoma	OV-IL15C plus EGFR-CAR-NK cells synergistically suppressed tumor growth and significantly improved survival compared with either monotherapy, correlating with increased intracranial infiltration and activation of NK and CD8+ T cells and elevated persistence of CAR-NK cells in an immunocompetent model. Collectively, OV-IL15C and off-the-shelf EGFR-CAR-NK cells represented promising therapeutic strategies for GBM treatment to improve the clinical management of this devastating disease (174).

### 5.1 Arming oncolytic virus with antibody and its derivatives

#### 5.1.1 OVs plus antibodies

Antibodies with different frameworks have been approved as cancer therapeutics; each has a different mode of action, such as blocking, neutralizing, and activating functions (159, 175, 176). These antibodies have been also coupled to various viruses such as Ad, MV, HSV, NDV, Reovirus, Vaccinia, and VSV in cancer combinational therapy (177–179), and some with promising results have been discussed below.

##### 5.1.1.1 OVs plus immune checkpoint inhibitors

It has been shown that the expression of inhibitory receptors on T-cells, including programmed cell death protein 1 (PD-1) and cytotoxic T-lymphocyte-associated antigen 4 (CTLA-4), and their binding to their ligands on the target cells (PDL-1 and B7, respectively) leads to aberrant activation of T-cells. In this regard, blockade of these negative regulators by ICIs can prevent T-cells suppression and improve their optimal activity in combating tumor cells (179). Nevertheless, the clinical response of ICIs correlates with pre-existing anti-tumor immune responses, such as an elevated number of tumor-infiltrating lymphocytes (TILs) and enough expression of immune checkpoints (ligands) on the tumor cells (45, 180). It has been shown that after viral infection, the expression of immune checkpoints upregulates on the surface of tumor cells, and accordingly, one of the most compelling combinational therapies for cancer would be OVs + ICIs (45, 181). In addition, another reason that makes the combination of OVs and ICIs attractive in the treatment of cancer is their different mechanism of action, which is an important parameter from the pharmacological point of view (45).

Zamarin et al. showed that localized OVT by NDV overcomes systemic tumor resistance to ICIs by inflaming the TME. They showed that I.T. administration of NDV not only increased infiltration of the lymphocytes into the injected tumor in the B16 melanoma mice models, but also the anti-tumor effect was observed in a distant tumor without any virus injection. Also, the localized administration of NDV in combinational with systematic administration of CTLA-4 blockade exhibited more efficient anti-cancer outcomes (182). In most studies for assessment of combinational OV-ICI therapy, fully humanized IgG antibody format has been used as ICIs; FDA-approved ICIs, especially those in clinical trials and has been discussed in detail elsewhere (178, 180). For example, although immune checkpoint inhibition is a logical therapeutic candidate against glioblastoma cells due to the increased expression of PD-1 on these cells (along with IL-10 and TGF-b), anti-PD-1 alone could not sufficiently eliminate tumor cells and there was a need for synergistic interactions with OVs. So, Saha et al. showed that a triple combination using anti-CTLA-4, anti-PD-1, and G47D-mIL12 (recombinant HSV virus) cured most mice in two glioma models. This approach not only treated mice, but also protected them against tumor re-challenge. The synergistic activity was associated with increased M1-like macrophages, T effector cells (CD4^+^ and CD8^+^), and decreased T regulatory (Treg) cells (183, 184). To emphasize the effect of combination therapy, it should be noted that while none of these agents were effective alone enough, they showed remarkable therapeutic effects in combinational strategy (184). It is worth noting that although Imlygic^®^ has been approved for adult patients with melanoma, there are clinical trials for the assessment of Imlygic^®^ in combination with ICIs for improving the treatment outcomes in melanoma and other cancer types (e.g., Pembrolizumab with Imlygic^®^ or Placebo in Unresected Melanoma, NCT02263508) (181, 185). Other combinations, including this OV and ICIs for triple-negative breast cancer with metastatic liver cancer and colorectal carcinoma are also being evaluated in clinical trials (186).

##### 5.1.1.2 OVs plus monoclonal antibodies other than ICIs

Apart from ICIs, some commercial mAbs with distinct mechanism of actions, have also been combined with OVs to improve the therapy outcome. In one study, the antitumor activity of cetuximab (an epidermal growth factor receptor inhibitor mAb) was assessed in combination with HSV. The result showed that combining cetuximab and HSV could improve distribution of the virus and lead to a synergistic antitumor effect in HT-29 tumor xenograft models (187). In another study, Zhang et al. demonstrated that combination of recombinant oncolytic HSV with Bevacizumab (BEV) (which is an antiangiogenic mAb approved for glioblastoma) in mice-bearing human GBM, was led to improvement of antiangiogenic effect of BEV while decreasing the tumor invasive-like phenotype induced by this drug (188).

#### 5.1.2 OVs plus nanobodies

Nanobodies are the smallest natural antigen-binding constructs with a single variable domain (VHH, ∼15kDa) as the antigen-binding region (189). Nanobodies have unique characteristics, such as easy selection by phage display, ease of manipulation, high stability in harsh conditions, and reaching and recognition of specific hard-to-access epitopes, making them more attractive in combination with other agents in cancer immunotherapy, including OVs (190, 191). For instance, due to the high complexity of glioblastomas and the low accessibility of therapeutic agents to their TME, the combination of viruses and nanobodies is a promising candidate for glioblastoma treatment. In a proof-of-concept study, Gil et al. used an anti-CXCR4 nanobody for retargeting oncolytic HSV toward CXCR4^+^ GBM cells. CXCR4 is overexpressed in various cancers, including glioblastoma, and usually correlates with a poor prognosis. The results of this study indicated that OVs plus nanobodies were highly encouraging for targeting GBM cells (192).

CD47 acts as a ”don’t eat me signal” to the immune system’s macrophages, making it a potential therapeutic target in some cancers. Different viruses have been engineered to express anti-CD47 antibodies (193) or nanobodies (194) to have a multifaced attack on the tumor cells. In a study, anti-CD47 nanobody-expressing adenovirus reprogramed tumor immune microenvironment and showed excellent anti-tumor immunity (194). This anti-CD47 oncolytic adenovirus could induce durable tumor suppression by changing the TME condition and increasing activated TILs in the tumor site. Systemic anti-tumor effects and memory immune cells were also observed after treatment by this recombinant virus (194).

#### 5.1.3 OVs plus bispecific or trispecific antibodies

Bispecific antibodies (bsAb) are constructs with two different antigen-binding sites with the aim of dual targeting (195). The coding sequence of bsAbs can be inserted into the viral genome to be expressed in the target tissue. Different viruses are engineered to this end, including Ad, HSV, MV and vaccinia (159, 196). Bi/trispecific antibodies can also be used in combination with viruses in various timings and dosages.

##### 5.1.3.1 OVs plus bispecific T-cell engagers

Viruses have been successfully combined with T-cell retargeting bsAb, also called Bispecific T-cell Engager or BiTe (197), to retarget T cells to the targeted tumor cells. In one study, a recombinant adenovirus encoding bsCD3-EpCAM bispecific antibody (bsAb) could effectively activate T-cells in malignant peritoneal and pleural exudates despite the immunosuppressive environment (198). Also, in another study, NDV-BiTe constructs (e.g., antiHN scFv/antiCD3 scFv and antiHN scFv/antiCD28 scFv) were successfully designed and expressed to be evaluated for thier remarkable potentials in tumor immunotherapy especially in breast and colorectal cancers (199, 200).

##### 5.1.3.2 OVs plus natural killer cell engagers

Emerging role of NKs cells in cancer therapy becomes clearer every day and its combination with virotherapy has accelerated the progress of therapeutic processes in cancer. Viruses can also be combined with bispecific NK engagers (201) to retarget NK cells to their targeted tumor cells.

As an examples for this type of combination, Bahrololoumi et al. constructed a bsAb (antiHN scFv/antiCD16 scFv) and a trispecific antibody(antiHN scFv/IL-15/antiCD16 scFv) to bind to the haemagglutinin neuraminidase (HN), a viral protein that is expressed on the surface of the NDV infected tumor cell, and the CD16 activating receptor on the surface of the NK cells for redirecting NK cells toward the tumor cells (201, 202). NDV-Ulster is a non-lytic strain of NDV that was used to inflame the tumoral microenvironment in this study, and also in NDV-based autologous tumor cell vaccines for stimulating the immune response in the patient’s body (93, 178, 203, 204).

##### 5.1.3.3 OVs plus trispecific antibodies

Trispecific antibody (Trike) is a single engineered antibody platform that recognizes and binds to three different targets and is expected to boost immune response significantly (205). There are different studies that combine these trifunctional engagers with OVs with the aim of cancer viro-immunotherpy. In one study, researchers constructed a trispecific immunocytokine (anti-NDV/IL2/anti-CD28) for efficiently targeting tumor cells. This trike could bind to the HN of the NDV infected tumor cells from one side and to the CD28 receptor on the T cells from the other side, while IL-2 promoted T cells function (206, 207). Ravirala et al, showed that combination of oncolytic HSV with bi/tri specific antibodies which could bind to the NKG2D and epidermal growth factor (EGF) from each side, while the trispecific one also contained IL-2 sequence, could significantly enhance infiltration and activation of NK and T cells in the tumor site (208). Various bi/trispecific antibodies that were combined with different viruses have been extensively reviewed elsewhere (209).

### 5.2 OVs plus CAR-T and CAR-NK cells

Chimeric antigen receptor (CAR)-T cells are T cells that have been genetically engineered to express an artificial receptor to direct them toward a specific target in an MHC-independent manner. The external domain of CAR-T cells consists of an extracellular target antigen binding domain which is usually a single-chain fragment variable (scFv) from a specific monoclonal antibody, attached to the transmembrane and signaling domains of this artificial receptor by a hinge (210). Although some scFv-based CAR-T cell products are currently approved by FDA for B-cell malignancies with encouraging results, this approach still faces some limitations, such as trafficking and tumor infiltration, antigen escape, immunosuppressive microenvironment, and CAR-T cell-associated toxicities (211). As a consequence, CAR-T cells do not exhibit profound anti-tumor effects in solid tumors. The use of VHH-based CAR-T cells (Nanobody CAR-T cells) may could resolve the abovementioned problems (212, 213). There are different antigens that are targeted through Nanobody-based CAR-T cells, such as vascular endothelial growth factor receptor 2 (VEGFR2) (214), human epidermal growth factor receptor 2 (HER2) (215), tumor-associated glycoprotein 72 (TAG‐;72) (216), prostate-specific membrane antigen (PSMA) (217–219), glypican 2 (GPC2), epidermal growth factor receptor (EGFR), B‐;cell maturation antigen (BCMA), PD‐;L1, and EIIIB (212, 220). It has been shown that the combination of CAR-T cells (scFv or VHH-based) with virotherapy can help CAR-T cells overcome their challenges in combat against solid tumors (such as immunosuppressive TME and heterogeneity of the antigens) and increase the immune response dramatically. For instance, Nishio et al. showed that armed oncolytic Ad (with RANTES and IL-15) could increase the efficacy of GD2 targeting CAR-T cell in a neuroblastoma solid tumor model (221). Furthermore, there is evidence that pre-treatment of solid tumors with OVs before the administration of CAR-T cells may lead to better ICD (222, 223). For example, combining recombinant oncolytic Ads containing a coding sequence of different cytokines, such as IL-2, RANTES and TNF-α, could lead to better accumulation and survival of CAR-T cells (223). Some of the combinations, such as Ad/HER-2 targeting CAR-T cell (NCT03740256) and VZV/GD2 targeting CAR-T cell (NCT01953900) therapy, are examples of such combinations being evaluated in clinical trials.

In the case of CAR-NK cells, in combination with HSV and to treat brain cancer metastases, EGFR CAR-NK cells were used intracranially in mice. This combination resulted in significantly longer survival of tumor-bearing mice when compared to monotherapies (224).

OVs attack and destroy tumor cells preferentially. Lysis of tumor cells releases neoantigen, PAMPs which trigger PRRs, which then produce inflammatory cytokines and antiviral type I IFNs. Viruses can activate cell death pathways, resulting in immunogenic cell death phenotypes such as necroptosis, pyroptosis, immunogenic apoptosis, and autophagic cell death. Antibodies that target cell surface indicators of immune cells (checkpoint inhibition), cancer cells (targeted therapy), or both (bispecific antibodies) are well-established in cancer therapy. combination of oncolytic viruses with antibody and CAR-T cells; CAR-T cells bind to the antigen on the surface of tumor cells and kill them, but they cannot migrate deeper into the dense tumor mass to remove antigennegative tumor cells. also, CAR-NK cells show more anticancer activity than CAR-T cells because they attach to stress ligands on the surface of tumor cells. The oncolytic virus attacks and destroys tumor cells, eliminating the tumor’s dense structure.

### 5.3 Other combinations with OVs

#### 5.3.1 OVs plus autologous DC or T cells

OVs can also be combined with cell therapy to treat different tumors, including solid tumors. For instance, the combination of NDV and dendritic cells (DC (as an exciting platform is being used in the IOZK clinic in Cologne Germany (Immun-Onkologisches Zentrum Köln). Their studies and practices in the IOZK indicated that DCs loaded with the lysate of NDV-infected tumor cells (viral oncolysate, VOL) triggered potent anti-tumor immunity by promoting the secretion of IFN-γ and IL-2 from T-cells. This combinational therapy is now available in the IOZK clinic and patients can benefit from the advantages of this kind of cancer combinational treatment (200, 225). Activation of naïve human T-cells by co-incubating with NDV-infected irradiated autologous tumor cells (ATV-NDV) which can be further modified with bi-specific or tri-specific antibodies can also offer a promising multimodal anti-cancer approach (226).

Altogether, strong evidence confirm that different therapeutic agents can have a measurable therapeutic effect in cancer treatment, but due to the specific and complex biology of cancer and its TME, the therapeutic outcome of these agents lonely, do not contribute to the final treatment of cancer patients. Thus, this is where rational combination therapy of these factors with each other, especially with OVs is much needed.

#### 5.3.2 OVs plus tumor-infiltrating lymphocytes

It has been demonstrated that weak functionality of natural TILs in the tumor site is strongly related to tumor progression (227). OVs can set the scene by inflaming the tumor microenvironment for better functionality of TILs. Feist et al, showed that local injection of poxvirus into a solid tumor in mice, could lead to activation and accumulation of TILs in the tumor site which had a low immunogenicity before virus infection (228). In another study, it was shown that virotherapy with a type of oncolytic adenovirus, could increase the TILs and significantly reduce the tumor size in the immunocompetent mouse model (229). It has also been shown that infecting the tumor with recombinant oncolytic HSV, could unleash the full potential of TILs which led to tumor regression and antitumor immunological memory (230).

## 6 Conclusions

OVT success, specifically FDA- and regional-approved OVs, has made waves in (pre)clinical areas, attracting both society and the scientific community’s attention. However, some of challenges have limited OVs application as immunotherapy, and their combination with other biotherapeutic platforms has been proposed in cancer therapy. To date, hundreds of combinations of OVs with other biotherapeutic platforms, including antibodies, nanobodies, ICIs, CAR-T cells, and DCs, have been investigated in clinical trials to understand which and how best to provoke anti-cancer immune responses. Some considerations could improve the efficacy of OVs, either as monotherapy or combination therapy. First, the dosage, targeted mechanisms, administration schedule, delivery technologies, and types of OVs could be considered because of their indispensable roles in the outcome of cancer immunotherapy and priming TME in combinational regimens. Second, understanding the interaction between immune cells/system, tumor cells/TME, and OVs and the combinational agents should help make new therapeutic combinations possible. Third, defining reliable biomarkers to distinguish “hot tumors” from “cold ones” can help scientists determine subsequent therapies. Finally, providing beneficial impacts of OVs and their combinational regimens on patients’ life quality requires the contribution of molecular biologists, pharmacologists, immunologists, and clinicians. Indeed, current clinical trials results can help scientists develop new systems of combination therapy and deliver innovative treatments to patients.

## Author contributions

ZS conceived the presented idea and SA developed the theory. MJ took the lead in writing the manuscript and she was in charge of overall direction and planning. MJ, MK and MBS wrote the manuscript with input from all authors. NH contributed to the writing of the results and to the editing of the manuscript. MAS and AA contributed to the final version of the manuscript. All authors discussed the results and contributed to the final manuscript

## Acknowledgments

We gratefully acknowledge the Immunology Department of Pasteur Institute of Iran for their technical assistance.

## Conflict of interest

Author MBS was employed by HUM Immune Biotech.

The remaining authors declare that the research was conducted in the absence of any commercial or financial relationships that could be construed as a potential conflict of interest.

## Publisher’s note

All claims expressed in this article are solely those of the authors and do not necessarily represent those of their affiliated organizations, or those of the publisher, the editors and the reviewers. Any product that may be evaluated in this article, or claim that may be made by its manufacturer, is not guaranteed or endorsed by the publisher.
